# Public Performance Metrics: Driving Physician Motivation and Performance

**DOI:** 10.5811/westjem.2020.1.41798

**Published:** 2020-02-24

**Authors:** Maxwell Y. Jen, Vy Han, Kathryn Bennett, Scott E. Rudkin, Andrew C. Wong, Erik D. Barton, Ronald Goubert

**Affiliations:** * University of California Irvine, Department of Emergency Medicine, Orange, California; † University of California Irvine, School of Medicine, Irvine, California

## Abstract

**Introduction:**

As providers transition from “fee-for-service” to “pay-for-performance” models, focus has shifted to improving performance. This trend extends to the emergency department (ED) where visits continue to increase across the United States. Our objective was to determine whether displaying public performance metrics of physician triage data could drive intangible motivators and improve triage performance in the ED.

**Methods:**

This is a single institution, time-series performance study on a physician-in-triage system. Individual physician baseline metrics—number of patients triaged and dispositioned per shift—were obtained and prominently displayed with identifiable labels during each quarterly physician group meeting. Physicians were informed that metrics would be collected and displayed quarterly and that there would be no bonuses, punishments, or required training; physicians were essentially free to do as they wished. It was made explicit that the goal was to increase the number triaged, and while the number dispositioned would also be displayed, it would not be a focus, thereby acting as this study’s control. At the end of one year, we analyzed metrics.

**Results:**

The group’s average number of patients triaged per shift were as follows: Q1–29.2; Q2–31.9; Q3–34.4; Q4–36.5 (Q1 vs Q4, p < 0.00001). The average numbers of patients dispositioned per shift were Q1–16.4; Q2–17.8; Q3–16.9; Q4–15.3 (Q1 vs Q4, p = 0.14). The top 25% of Q1 performers increased their average numbers triaged from Q1–36.5 to Q4–40.3 (ie, a statistically insignificant increase of 3.8 patients per shift [p = 0.07]). The bottom 25% of Q1 performers, on the other hand, increased their averages from Q1–22.4 to Q4–34.5 (ie, a statistically significant increase of 12.2 patients per shift [p = 0.0013]).

**Conclusion:**

Public performance metrics can drive intangible motivators (eg, purpose, mastery, and peer pressure), which can be an effective, low-cost strategy to improve individual performance, achieve institutional goals, and thrive in the pay-for-performance era.

## INTRODUCTION

As healthcare reimbursement shifts from “fee-for-service” to “pay-for-performance,” strategies designed to incentivize improved physician performance must evolve in tandem. Across industries, financial incentives and disciplinary threats are frequently used; however, prior studies have demonstrated that these so-called extrinsic motivators lead to worsened performance for any task requiring even rudimentary cognitive ability.^1^ In contrast, prior studies have also shown that intrinsic motivation—personal motivators based on a sense of pride, accomplishment, and mastery rather than a desire for money or fear of reprisal—is much more effective at driving performance outcomes for highly cognitive tasks. In fact, as open disclosure of healthcare performance outcomes becomes more common as a health policy tool,^2^ advocates cite that public disclosure of poor individual physician performance directly drives positive physician behaviors that result in improved performance and patient outcomes.^3^

Existing evidence on the impacts of publicly reporting individual performance outcomes offers varied results. One study describing Los Angeles schoolteacher performance concludes that openly disclosing performance outcomes to one’s peers and the public drove improvement by appealing to the teachers’ desire to protect their reputations and allowing them to benchmark their individual effectiveness vs their peers.’^4^ However, the study also found deleterious effects in one subgroup that experienced feelings of anger and embarrassment, which ultimately hampered intrinsic motivation.

To our knowledge, there are no studies to date that examine the outcomes of reporting individual performance outcomes of physicians. This study aimed to determine whether open reporting of a select set of performance metrics at the individual physician level could influence individual behavior and drive future improvements in those measured metrics. Specifically, we examined performance within an emergency department (ED) physician-in-triage (PIT) system. In our study, PIT physicians’ only tasks are to sort patients, based on a history and physical exam, into two groups—those that require further evaluation and stabilization vs those who require minimal to no further evaluation—and discharge patients in the latter group. PIT performance was subsequently openly published to all PIT physicians.

## METHODS

This was a retrospective review of individual emergency physicians’ (EP) triage-system performance data from a single-site, academic tertiary-care center’s ED over a 12-month period from September 1, 2015, through August 31, 2016. During this period, total ED census was 50,140 patients. The ED triage system consisted of three patient evaluation spaces and was staffed with one of 24 board-certified or board-eligible emergency medicine (EM) attending physicians and two to three EM nurses with 14 total hours of physician coverage per day from 10 am to midnight. Each physician’s “triage shift” consisted of seven hours patient evaluation and one hour of charting. On average, EM attending physicians worked 25% of their scheduled shifts as PITs.

The EPs were informed that the triage system would have two goals: 1) Based on a limited history and exam, rapidly identify and sort (ie, “triage” patients into either a moderate-to-high acuity group that required moderate-to-extensive evaluation and management or into a low-acuity group that required little to no management); and 2) disposition (which was primarily quantified as discharges from the ED but also included “direct” admits to the main hospital) of all patients in the low-acuity group, as appropriate. Patients sorted into the moderate-to-high acuity group were subsequently assigned to another EP for further care. For patients designated as moderate-to-high acuity, the EP would document a 1–3 sentence note describing the initial impression and order labs, imaging, and or medications via computerized order entry (COE) system. For patients who could be rapidly dispositioned, the EP would chart the full ED note and order all studies, medications, and prescriptions via COE. Based on these goals, we tracked the two performance measures for each individual physician: 1) mean number of patients triaged per shift; and 2) mean number of patients dispositioned per shift.

**Population Health Research Capsule**

What do we already know about this issue?

Evidence on public reporting to drive improvements has been mixed. This is the first study to analyze whether publicizing individual metrics affects emergency department (ED) triage performance.

What was the research question?

Does publicly displaying physician performance metrics influence the individual physician’s future triage performance?

What was the major finding of the study?

Displaying public performance metrics correlated with a statistically significant increase in the average patients triaged per hour.

How does this improve population health?

Improved triage performance has been shown to lead to improvements in door-to-physician time, length of stay, and left without being seen visits, despite increasing ED volume.

Before data collection for this study began, the PIT system had been in place for three months to allow for any learning curve with this change to physician practice. We extracted these metrics for individual PIT physicians from the electronic medical record system without patient identifiers. Performance metrics for all 24 PIT physicians were reported on a quarterly basis openly to the PIT physician group; the average and standard deviation for each metric was also reported to the group. Performance metrics were not linked to any material or financial incentive or disincentive. While goals were reiterated quarterly and physicians were encouraged to practice as they deemed safe and appropriate, no specific guidance or remediation strategies were created, administered, or required for any physician at any point before, during, or after the study period; physicians were free to apply or disregard the data as they wished.

After the study period, we examined the group’s average quarterly performance for all four quarters and trended the comparison over time. Using Student’s t-test, we compared the group’s average performance in the first quarter of the study vs the final quarter. Given the possibility that particular subgroups’ performance would evolve in different ways, we also compared the performance of the top and bottom quartile of performers.

## RESULTS

**Figure 1:**
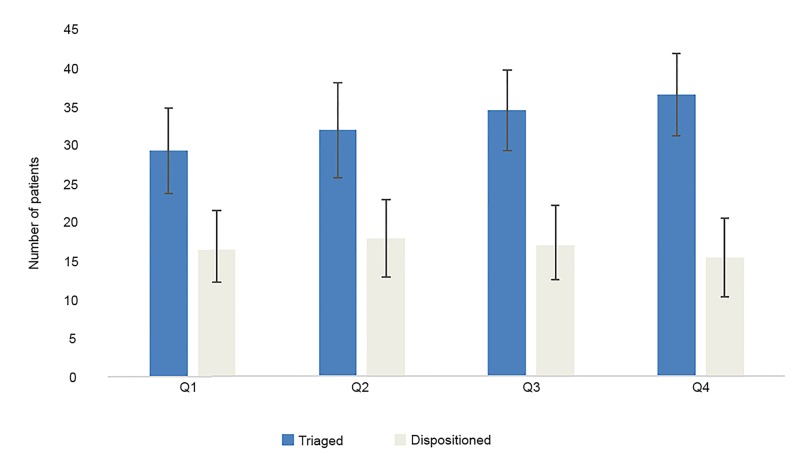
Average number of patients triaged vs dispositioned by yearly quarter for all providers ± standard deviation.

During the first quarter, the average number of patients triaged and dispositioned over seven-hour shifts by all physicians were 29.1 (±5.5) and 16.4 (±4.3), respectively. For the second quarter, the average number of patients triaged and dispositioned were 31.9 (±6.1) and 17.8 (± 5.0), while for the third quarter, the average number of patients triaged and dispositioned were 34.3 (± 5.2) and 16.9 (±4.5), respectively. In the final quarter of the study period, the average number of patients triaged was 36.5 (±5.3), and the average number of patients dispositioned was 15.3 (±5.0). Results are summarized graphically in Figure 1.

After Q1, the top and bottom 25% quartile groups by number of patients triaged were identified. These groups were determined a priori before analysis in order to avoid Type I error. The top 25% of Q1 performers increased their average numbers triaged from Q1–36.5 to Q4–40.3 (ie, a statistically insignificant increase of 3.8 patients per shift [p = 0.007; Table 2]). The bottom 25% of Q1 performers, on the other hand, increased their averages from Q1–22.4 to Q4–34.5 (ie, a statistically significant increase of 12.2 patients per shift [p = 0.0013; Table 2]). The number of patients dispositioned in Q1 vs Q4 was determined not to be statistically significant (a decrease of 1.1 patients per shift; p = 0.142; Table 1).

## DISCUSSION

Measuring physician performance is a difficult task as many variables are involved to performing the job of a physician. Physicians have historically been reluctant to have their efficiency of patient throughput objectively measured, given the many confounders that affect their daily decision-making.^13^ These variables include,the following: input; lengthy or inefficient admission processes; patient disease characteristics; and system-level factors (eg, ED staffing, difficulty getting timely consultations, a lack of available inpatient beds, timeliness of labs or radiology interpretations).^14^ Furthermore, many contend that elements involved in patient care, such as compassion and communication, are difficult for objective data capture yet contribute meaningfully to outcomes of morbidity and mortality.^15^

Increasing ED visits and concomitantly ED crowding represent a major challenge for healthcare systems across the country.^16^ The increase in wait times before patients are seen by a physician has led many facilities to adopt a PIT system as a strategy to alleviate ED crowding by improving throughput. PIT systems target and reduce initial long wait times for patients by ensuring rapid evaluation by a physician upon arrival to the ED. Orders are expedited with critically ill patients immediately identified and sent back to the main ED and low- acuity patients rapidly discharged. PIT programs have been proven to provide sustainable improvements in ED performance metrics such as including door-to-physician time, length of stay, and left without being seen visits, despite increasing ED volume.^17^

The results of this study demonstrate that simply displaying attending physician performance metrics at quarterly meetings led to an increase in patients triaged per hour. Furthermore, the increase was most notable and statistically significant for the bottom 25^th^ percentile of attending physicians, a cohort that improved their number of patients triaged per hour by 12.2 patients, or 54% quarter-over-quarter. Although not statistically significant, there were still improvements in patients triaged per hour in the top 25^th^ percentile performers of attending physicians, a cohort that was already outperforming its peers. This cohort increased the average patients triaged per hour by 3.8, a 10.3% quarter-over-quarter increase.

An increase in the number of patients triaged per hour has been shown to reduce waiting times and length of stay in the ED.^17^ While the significant investment required for designing, implementing, and evaluating the development of a PIT system must be considered, the long-term gains in various ED metrics may offset the upfront cost. Furthermore, once a triage system is established, publicly publishing triage performance metrics does not require any increase in resources, resulting in arguably only upside potential.

Our results align with much of the current literature regarding workplace motivation, finding that workers are rewarded for measurable performance improvement. While the logic behind extrinsic motivators (eg, more money for better performance) is intuitive, other studies^18^ concerning workers who perform higher level cognitive tasks, such as physicians, have shown that the best use of money is to pay workers just enough to take the issue of money off the table. Once this is done, there appear to be three factors, so-called intrinsic motivators, that lead to better performance when performing tasks that require higher-level of thinking: 1) autonomy, the ability to self-direct; 2) mastery, the desire to be the best at our artform or tasks; and 3) purpose, the idea that what we do is important and connected to our inner belief system. Publishing performance metrics while simultaneously allowing physicians to maintain their autonomy (physicians were allowed to practice how they desired), achieve mastery (bottom-performing physicians, known to all as laggards in the cohort, dramatically improved their ability and performance relative to their peers), and purpose (physicians were informed that this was an important group goal), is a simple yet powerful solution to improve motivation and performance.

Within healthcare, prior studies have only examined the effects of reporting performance at the institutional level with highly variable outcome measures and mixed results^5^,^6^,^7^,^8^ Interestingly, several studies demonstrated that public performance reporting had a greater effect on quality improvement than traditional performance evaluation alone and suggest that public performance reporting stimulates additional quality improvement activity,^9^,^10^,^11^ which then correlated with increased patient satisfaction and care outcomes.^12^

## LIMITATIONS

There were some limitations to this study. First is the question of whether performance increased solely due to the psychologically motivating effect of the published performance data or physicians improved simply due to practice. We believe that this effect was mitigated by the fact that the PIT system had already been operational for several months prior to the start of the study, which should have been adequate to adjust to any learning curve. Second, this study was performed at a single-center, academic tertiary care center and not a community ED where other factors may affect how physicians perform. There were a small number of attending physicians in this study, a total of 24. We did not control or analyze for volume of patients presenting during a given shift to the triage physician. It is possible, although unlikely, that arrival volume could affect triage per hour numbers in a systematic way.

Additionally, we did not collect or analyze outcome data for patients triaged by the group or individuals. Speed could conceivably have a negative effect on quality or cost. Finally, institutional culture plays a role in terms of how such a study is received among their physician staff. Like most real-world processes, the University of California, Irvine ED triage system constantly evolved in real-time. It would be beneficial to determine whether this study’s findings could be duplicated in EDs with differing triage systems or for other ED performance indicators such as computed tomography utilization rates, length of stay, or patients per hour.

## CONCLUSION

Most business organizations now look for a transcendent purpose within an organization to help foster a sense of contribution from their workforce. This study shows that public performance metrics had a correlation with increased performance among physicians. Public performance metrics can encourage mastery within one’s profession by demonstrating what was possible within the top 25% of performers. By reinforcing autonomy and allowing physicians to practice the way they prefer to, we increase engagement within the work force. This study also challenged the traditional belief that financial incentives are tied to increase in production. The lack of a financial incentive within this study did not deter improvement in performance. This study demonstrates it is possible to increase and improve performance without increasing departmental operational cost.

## Figures and Tables

**Table 1:** Overall triage and disposition performance Q4 vs Q1.

	Q1	Q4	95% Confidence interval (CI) for difference Q4 vs Q1		P-value
Number of patients dispositioned per shift	16.4	15.3	↓	1.1 patient /shift; CI: -3.8 to 1.7	0.1424
Number of patients triaged per shift	29.2	36.5	↑	7.3 patients/shift; CI: 4.18 to 10.5	<0.05

**Table 2:** Triage and disposition performance by top and bottom quartile performers Q4 vs Q1.

	Q1	Q4	Difference and confidence interval (CI) for Q4 vs Q1		P-value
Number of patients patients triaged per shift by top 25% performers	36.5	40.3	↑	3.8 patients/shift; CI: 0.05 to 7.5	<0.05
Number of patients triaged per shift by bottom 25% performers	22.4	34.5	↑	12.2 patients/shift; CI: 8.4 to 15.9	<0.05
